# Vaccine Hesitancy Among Caregivers in China: Associations of Misinterpreted AEFI, Compensation Awareness, and Institutional Trust

**DOI:** 10.3390/vaccines14070577

**Published:** 2026-06-30

**Authors:** Binyue Xu, Qing Wang, Jiawei Xu, Ningpei Bai, Chunbei Zhou

**Affiliations:** Chongqing Center of Disease Control and Prevention (Chongqing Academy of Preventive Medicine), Chongqing 400016, Chinagongweibainp@163.com (N.B.)

**Keywords:** vaccine hesitancy, adverse events following immunization (AEFI), health communication, caregivers

## Abstract

Background: Vaccine hesitancy remains a major global public health challenge despite high immunization coverage. While concerns about adverse events following immunization (AEFI) are widely recognized, limited attention has been paid to caregivers’ ability to distinguish vaccine-related adverse reactions from coincidental illnesses, as well as to the associations of compensation awareness and institutional trust with vaccination decisions. Methods: A cross-sectional survey was conducted between January and March 2025 among 3497 caregivers of children aged 0–6 years using multistage random sampling. Data were collected on AEFI knowledge (including understanding of coincidental illnesses), compensation mechanism awareness, institutional trust, and vaccination behaviors. Vaccine hesitancy was assessed using a self-reported measure and the WHO SAGE Vaccine Hesitancy Scale (VHS). Multivariable linear regression models were used to estimate factors associated with hesitancy scores. Results: The prevalence of vaccine hesitancy was 8.6%, with a mean VHS score of 23.2 ± 4.2. Although general awareness of AEFI reached 90.0%, correct understanding of coincidental illnesses was only 25.7%. Higher AEFI knowledge and compensation awareness were associated with lower hesitancy scores, whereas distrust in compensation mechanisms and perceived vaccine unsafety were associated with higher hesitancy. Coverage of national immunization program (NIP) vaccines exceeded 90%, while uptake of self-paid vaccines varied from 46.7% to 89.7%. Conclusions: Vaccine hesitancy among caregivers was associated with cognitive understanding, economic considerations, and institutional trust. Limited understanding of coincidental illnesses may be associated with increased concerns about vaccine safety, while compensation awareness and institutional trust were associated with lower levels of vaccine hesitancy. Strengthening communication on AEFI causality and improving transparency of compensation policies may help reduce vaccine hesitancy and support immunization programs in settings with mixed public–private financing systems.

## 1. Introduction

**Figure 1:**
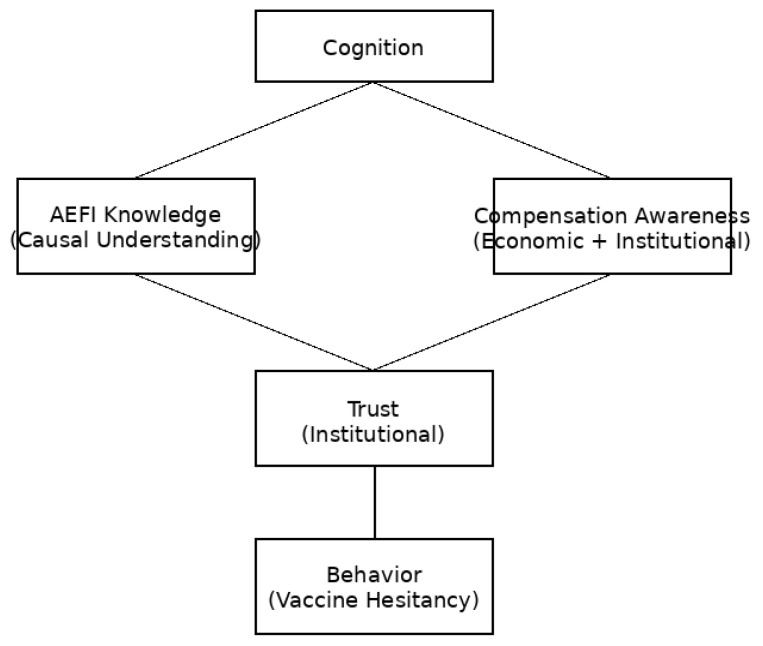
Hypothesized factors associated with vaccine hesitancy within the cognition–trust–behavior framework.

Vaccination is one of the most cost-effective public health interventions to reduce the incidence and mortality from children’s infectious diseases [1]. The World Health Organization (WHO) notes that vaccines prevent millions of deaths caused by vaccine-preventable diseases every year [2]. Nevertheless, vaccine hesitancy has been listed as one of the top ten global health threats in recent years, and its adverse impacts on the National Immunization Program (NIP) have become increasingly prominent [3]. In China, the coverage of NIP vaccines has remained at a high level, while public attention has gradually shifted to vaccine safety, adverse events following immunization (AEFI), and supporting compensation mechanisms [4]. Previous studies have confirmed that worries over AEFIs are strongly associated with vaccine hesitancy, partly due to insufficient public health literacy and cognitive biases toward vaccination risks [5]. Furthermore, since non-NIP vaccines require self-payment, economic costs and perceived risks interact closely, jointly serving as critical predictors of vaccination uptake [6]. Unlike the single compensation model adopted by most high-income countries, several Chinese regions including Chongqing have established a dual AEFI compensation system, which comprises basic government financial indemnity and supplementary commercial insurance voluntarily purchased by guardians. Although this dual structure is designed to deliver more comprehensive financial safeguards, public awareness of the relevant policies varies widely across populations. To date, limited research has investigated how this unique institutional arrangement affects vaccine hesitancy, especially in western Chinese regions with prominent urban–rural gaps. In addition, few domestic studies integrate AEFI knowledge, compensation awareness, and institutional trust within a unified analytical framework, particularly using large-scale data from western China [7]. Moreover, existing research generally treats AEFI cognition as a homogeneous construct, with limited attention to the distinction between general awareness and causal understanding—such as the ability to correctly identify coincidental illnesses that are temporally, but not causally, related to vaccination. In this study, a coincidental illness refers to a health event that occurs after vaccination in temporal sequence but is not causally attributable to the vaccine. In addition, the moderating effect of AEFI compensation policies on public risk perception and institutional trust remains under-explored. The present study was informed by an extended trust–cognition–behavior framework, which proposes that health-related behaviors are associated with both cognitive factors and trust in relevant institutions. Within this framework, knowledge and understanding of vaccine-related issues, including AEFI and coincidental illness, represent cognitive dimensions, whereas confidence in vaccine-related institutions and compensation arrangements reflects institutional trust. These factors may be associated with caregivers’ vaccination attitudes and decisions, providing a useful conceptual framework for examining vaccine hesitancy (Figure 1).

As a highly populous municipality in western China with significant urban–rural disparities, Chongqing is a typical representative of the country’s dual urban–rural structure providing ideal research setting to explore factors associated with vaccine hesitancy. Based on an extended trust–cognition–behavior theoretical framework, this study systematically assessed caregivers’ knowledge of AEFI, with particular attention paid to their understanding of coincidental illnesses, as well as their awareness of relevant compensation mechanisms. It also examined vaccine hesitancy and childhood vaccination behaviors among caregivers of young children. Collectively, the findings of this research may provide evidence to support vaccine-related risk communication strategies and improvements in compensation policy communication, thereby contributing to efforts aimed at maintaining vaccine confidence and vaccine uptake in Chongqing and similar settings. We hypothesized that clearer understanding of AEFI, particularly regarding coincidental illness, as well as greater awareness of compensation mechanisms and higher institutional trust, would be associated with lower vaccine hesitancy.

## 2. Methods

### 2.1. Study Subjects and Sampling Methods

A cross-sectional survey was conducted between January and March 2025 among guardians of children aged 0–6 years in Chongqing, China. Based on the results from the pilot study, the rate of hesitancy (P) was estimated at 7%, with an allowable error of 0.1P and a significance level of 0.05, and the required sample size was calculated as 2269. With a conservative estimate of a 15% non-response rate, the planned sample size was 2669 individuals. Eligible participants were caregivers aged 18 years or older who were the primary caregivers of children aged 0–6 years, were able to understand and complete the questionnaire independently, and provided informed consent. Caregivers were excluded if they were unable to complete the questionnaire independently because of cognitive or communication difficulties, declined participation, or submitted duplicate responses.

**Figure 2:**
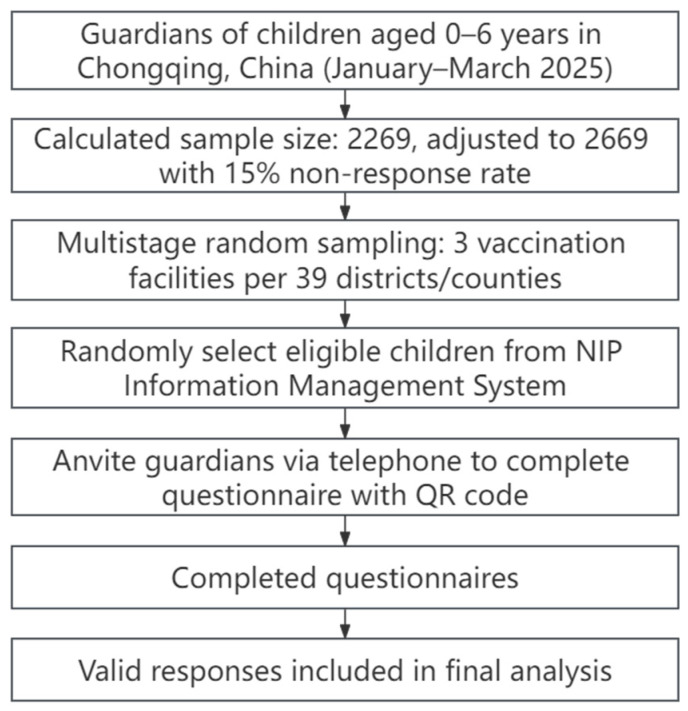
The flow of participant recruitment and inclusion.

A multistage random sampling approach was used. Three vaccination facilities were randomly selected from each of Chongqing’s 39 districts and counties. Eligible children aged 0–6 years were then randomly sampled from the NIP Information Management System using simple random sampling. Child guardians were invited to vaccination clinics via telephone appointments and completed the questionnaire independently by scanning a QR code on their mobile phones under one-on-one investigator guidance. Caregivers who were unable to complete the questionnaire independently or declined to participate were excluded. There were no substantial missing data. A flow diagram of participant recruitment is shown in Figure 2.

Several measures were taken to reduce potential bias. Standardized questionnaires and trained investigators were used to minimize information bias. Random sampling from the immunization registry helped reduce selection bias. In addition, anonymous data collection may have reduced social desirability bias.

### 2.2. Questionnaire

The questionnaire was reviewed and validated by NIP experts. It covered region type, demographic characteristics of children and their guardians, history of vaccine hesitancy and refusal, history of suspected AEFI in children, knowledge of AEFI and insurance compensation, attitudes toward vaccination, approval of the current vaccine injury compensation mechanism, and childhood vaccination status. The AEFI compensation mechanism refers to the administrative and financial arrangements established to provide compensation for vaccine-related injuries in accordance with national and local regulations. Approval of the current AEFI compensation mechanism was used as an indicator of institutional trust. The sections on AEFI knowledge and insurance compensation knowledge contained 5 and 8 items, respectively. Correct responses to ≥3 items for AEFI knowledge and ≥5 items for compensation knowledge were defined as adequate awareness. As no validated thresholds are currently available for these measures, a cutoff corresponding to approximately 60% of the maximum possible score was adopted, consistent with previous vaccination knowledge and health literacy research. In addition, a 60% threshold was adopted because it is commonly used in public health practice to identify populations with insufficient knowledge and to inform targeted intervention strategies.

Two complementary measures of vaccine hesitancy were used. The self-reported willingness question was used to estimate the prevalence of vaccine hesitancy, whereas a scale was used to assess the degree of vaccine hesitancy as a continuous measure. For the binary classification of vaccine hesitancy, participants responded to the question “Are you currently willing to take your child to be vaccinated at a vaccination clinic?” Responses of “uncertain”, “unwilling”, or “very unwilling” indicated vaccine hesitancy. For a continuous objective assessment, the WHO Strategic Advisory Group of Experts on Immunization (SAGE) 2015-recommended Vaccine Hesitancy 5-Point Likert Scale Questionnaire (VHS) was used [8]. The scale included 10 items, with responses rated as strongly disagree, disagree, neutral, agree, and strongly agree, scored 1–5 or 5–1 reversely, resulting in a total score ranging from 5 to 50. Higher scores indicated a higher level of vaccine hesitancy [9,10]. A pilot survey confirmed good reliability and validity of the questionnaire: Cronbach’s *α* = 0.810, KMO = 0.900, and Bartlett’s test of sphericity *p* < 0.001. Exploratory factor analysis extracted 2 common factors, with a cumulative variance contribution rate of 68.31%.

Non-NIP vaccines refer to voluntarily purchased vaccines not included in China’s national immunization program, with out-of-pocket costs borne by caregivers. Childhood vaccination status included NIP vaccines, namely hepatitis B vaccine, measles-containing vaccine, diphtheria–tetanus–pertussis (DTP) combined vaccine, and polio vaccine, and non-NIP vaccines, namely *Haemophilus influenzae* type b (Hib) vaccine, enterovirus 71 (EV71) inactivated vaccine, 13-valent pneumococcal polysaccharide conjugate vaccine (PCV13), varicella vaccine, rotavirus vaccine, and influenza vaccine.

### 2.3. Statistical Analysis

SPSS 25.0 was used for statistical analysis, including calculation of awareness rates of AEFI and insurance compensation knowledge, vaccine hesitancy rate, and mean vaccine hesitancy score among child guardians. Univariate analysis of factors associated with vaccine hesitancy scores was performed using the *t*-test or analysis of variance (ANOVA). Independent variables were meticulously screened following the principle of combining a statistical benchmark of *p*  ≤  0.2 in univariate test results and professional significance. Relevant variables were then included in the multivariate analysis. Because the VHS total score represents a composite measure derived from multiple Likert-scale items and demonstrated an approximately normal distribution, it was treated as a continuous variable. Therefore, multivariable linear regression was used to retain the continuous nature of VHS scores and improve statistical efficiency. The variance inflation factor (VIF) of each variable was calculated to diagnose multicollinearity, with VIF < 3 for all variables indicating no significant multicollinearity. No subgroup or interaction analyses were performed.

### 2.4. Ethics Statement

This study received approval from the ethics committee of the Chongqing Center for Disease Control and Prevention (KY-2023-021-1, approved on 15 June 2023; KY-2024-025-1, approved on 4 September 2024). Informed consent was obtained from all participants at the beginning of the survey.

## 3. Results

### 3.1. General Characteristics of Study Subjects

To enhance the representativeness of the data and prevent a shortage of valid samples arising from invalid questionnaires, we prospectively expanded the actual survey sample size in advance. In total, 3497 valid questionnaires were collected, with no substantial missing data for the main variables. The male-to-female ratio of respondents was 1.05:1 (1790/1707). Urban and rural household registration accounted for 71.1% and 24.9%, respectively. Respondents aged < 30 years, 30–39 years, and ≥40 years accounted for 33.8%, 56.2%, and 10.0%, respectively. Educational level was primary school or below (4.8%), secondary school or junior college (53.6%), and bachelor’s degree or above (41.6%). Children aged 0–1 year and 2–5 years accounted for 43.6% and 56.5% of surveyed children, respectively.

### 3.2. Knowledge of AEFI and Insurance Compensation

Among respondents, the awareness rates of knowledge related to AEFI and AEFI insurance compensation ranged from 25.7% to 97.7% and from 55.9% to 81.9%, with overall awareness rates of 90.0% (3148 participants) and 65.0% (2274 participants), respectively. Scores ranged from 0 to 5 (median: 4, interquartile range: 3–4) for AEFI knowledge, and from 0 to 8 (median: 7, interquartile range: 3–8) for AEFI insurance compensation knowledge (Table 1).

### 3.3. Actual Childhood Vaccination Behavior

Among age-eligible children surveyed, the coverage rates of NIP vaccines were all above 90%, including 96.1% (2328/3143) for measles-containing vaccine, 99.09% (3374/3405) for DTP vaccine, 90.08% (3095/3436) for hepatitis B vaccine, and 99.41% (3379/3399) for polio vaccine. The coverage rates of non-NIP vaccines varied widely: 57.2% (1956/3422) for Hib vaccine, 46.7% (1565/3354) for PCV13, 82.9% (2641/3186) for EV71 inactivated vaccine, 89.7% (2553/2845) for varicella vaccine, 61.0% (2046/3353) for rotavirus vaccine, and 70.5% (2224/3156) for influenza vaccine. And reasons for guardians refusing childhood vaccination included concerns about vaccine safety (47.9%, 23/48), belief that vaccination was ineffective (37.5%, 18/48), long distance to vaccination facilities (25.0%, 12/48), and advice from clinicians that vaccination was unnecessary (14.5%, 7/48).

### 3.4. Vaccine Hesitancy and Its Influencing Factors

Based on the self-reported willingness question, the overall vaccine hesitancy rate among respondents was 8.6% (301/3497; 95% CI 7.68~9.54), and the mean VHS score was 23.2 ± 4.2 (range 10–43). Normality testing confirmed an approximately normal score distribution, justifying the use of linear regression models.

Variables with *p* ≤ 0.2 in univariate analysis were included in multivariate regression, and multicollinearity was excluded. Multivariate linear regression analysis showed that higher vaccine hesitancy was independently associated with rural residence (*β* = 0.37, 95% CI: 0.05~0.69), not having attended parent classes (*β* = 0.54, 95% CI: 0.27~0.80), history of vaccine hesitancy (*β* = 1.65, 95% CI: 1.31~1.99), history of vaccine refusal (*β* = 1.04, 95% CI: 0.65~1.43), perception that childhood vaccines were “somewhat unsafe/unsafe” (*β* = 2.30, 95% CI: 1.92~2.68), disagreement with diagnoses by the AEFI investigation and diagnosis panel (*β* = 0.62, 95% CI: 0.29~0.95), and distrust in the AEFI compensation mechanism (*β* = 1.38, 95% CI: 1.01~1.75).

Lower vaccine hesitancy was associated with monthly household income of 10,000–19,000 yuan and ≥20,000 yuan (*β* = −1.08, 95% CI: −1.67~−0.49; *β* = −1.26, 95% CI: −2.28 ~−0.28), child aged 3–5 years (*β* = −0.56, 95% CI: −0.84~−0.27), higher AEFI knowledge score (*β* = −1.14, 95% CI: −1.41~−0.81), higher AEFI insurance compensation knowledge score (*β* = −2.06, 95% CI: −2.54~−1.58), and no out-of-pocket medical expenses in the event of AEFI (*β* = −0.76, 95% CI: −1.07~−0.46) (Table 2 and Supplementary Table S1).

With this multivariate linear regression framework, positive *β* values correspond to higher VHS scores (greater vaccine hesitancy) compared with the reference group, while negative *β* values signify lower VHS scores. Each *β* coefficient quantifies the average adjusted difference in VHS total scores associated with the corresponding predictor, after controlling for all other covariates entered into the model.

## 4. Discussion

This study provides population-based evidence on vaccine hesitancy among caregivers in China and highlights the independent correlations of cognitive understanding, economic considerations, and institutional trust with vaccine attitudes. It contributes to the literature by distinguishing between general AEFI awareness and understanding of coincidental illnesses, while simultaneously examining compensation awareness and institutional trust within the same study population. The overall prevalence of vaccine hesitancy was 8.6%. Although this rate is lower than those reported in some international studies, it still indicates a certain proportion of latent vaccine hesitancy against the backdrop of high coverage rates for national immunization program vaccines in China. This finding is generally consistent with recent studies in China but lower than rates reported in some European and American countries [3,11], which may be attributed to the high accessibility of China’s immunization program system and strong policy promotion. Meanwhile, global vaccine confidence has fluctuated considerably in the post-pandemic era, and vaccine hesitancy remains an ongoing public health concern [2]. Such potential hesitancy cannot be overlooked and warrants sufficient attention. Although conducted in Chongqing, a setting characterized by pronounced urban–rural disparities, high immunization coverage, and a dual public–private compensation system, these findings may have broader relevance for regions with similar health system structures and mixed vaccine financing mechanisms.

A key finding is the gap between general awareness of AEFI and the understanding of coincidental illnesses. The survey revealed that the overall awareness rate of AEFI was relatively high, at 90.0%. Most guardians were able to recognize common adverse reactions and basic vaccination protocols, including key requirements such as the 30 min on-site observation period after vaccination to ensure prompt resuscitation in case of acute anaphylactic shock. However, understanding of the concept of coincidental illnesses was notably poor, with an awareness rate of only 25.7%, indicating a substantial gap in caregivers’ understanding of AEFI causality and vaccine safety concepts, consistent with previous studies reporting challenges in public understanding of vaccine-related risks and adverse event attribution [5,6,12]. This finding suggests that many caregivers may have difficulty distinguishing causal relationships from temporal correlations in vaccine-related adverse events [13,14]. Such cognitive biases tend to amplify public perceptions of vaccine risks, thereby intensifying vaccine hesitancy [15]. Misunderstanding of coincidental illnesses was associated with greater vaccine hesitancy. This finding may reflect difficulties in distinguishing temporal coincidence from causal relationships, which could contribute to increased concerns about vaccine safety. These results suggest that vaccine-related education should place greater emphasis on explaining AEFI causality rather than focusing solely on general vaccine safety information. Instead, there is a greater need to strengthen the public’s ability to interpret risks and establish a scientific cognitive framework. Accordingly, future vaccine-related health education should focus on popularizing specialized content such as AEFI classification and causal judgment principles, rather than only general vaccine safety promotion, to help the public develop a rational understanding of vaccination.

Beyond cognitive factors, this study highlights the associations among compensation awareness, institutional trust, and vaccine hesitancy. Compared with knowledge of AEFI, guardians’ overall awareness of AEFI-related insurance and compensation was only 65.0%, with substantial disparities across different items. In particular, understanding of supplementary insurance coverage—such as compensation for lost work time and AEFI related to non-NIP vaccines—was severely inadequate, indicating clear blind spots in current policy communication. Multivariate analysis showed that higher compensation awareness was associated with lower vaccine hesitancy (*β* = −2.06, 95% CI: −2.54~−1.58), while distrust in the compensation system was associated with higher vaccine hesitancy (*β* = 1.38, 95% CI: 1.01~1.75). These findings are consistent with previous studies suggesting that institutional trust is related to vaccine hesitancy [16,17]. Furthermore, a recent systematic review noted that financial compensation or incentives only function effectively when the public holds high trust in relevant institutions [18]. However, because of the cross-sectional design, the direction of these relationships cannot be determined. Future longitudinal studies are needed to further explore how compensation awareness and institutional trust relate to vaccination attitudes over time.

Regarding vaccination behavior, this study found that the coverage rate of NIP vaccines remained high, all above 90%, whereas coverage of non-NIP vaccines varied widely, ranging from 46.7% to 89.7%. Notably, coverage of the PCV-13 was below 50%, suggesting that economic cost and risk perception remain major barriers to public uptake of non-NIP vaccines. This pattern aligns with global evidence that vaccination decisions involving out-of-pocket costs are particularly sensitive to risk–cost trade-offs, highlighting the importance of both perceived risk and financial considerations in vaccination decisions beyond the Chinese context [19]. Among the reasons guardians refused vaccination, concerns about vaccine safety (47.9%) and doubts about vaccine effectiveness (37.5%) ranked as the top two. This suggests that concerns about vaccine safety and effectiveness were commonly reported among caregivers who refused vaccination [12,20,21]. These results imply that when promoting non-NIP vaccines, equal attention should be paid to risk communication and economic accessibility.

Multivariate analysis identified several factors associated with vaccine hesitancy. Perceived vaccine unsafety showed one of the strongest associations with higher vaccine hesitancy (*β* = 2.30, 95% CI: 1.92~2.68), representing one of the strongest factors associated with higher hesitancy scores. Guardians with a history of vaccine hesitancy or refusal were also more likely to report higher hesitancy. This phenomenon is supported by previous research, including longitudinal studies indicating that parental decisions about childhood vaccination are often formed prior to childbirth, persist over time, and influence subsequent vaccination behaviors [22]. Regarding institutional trust, guardians who disagreed with AEFI diagnostic conclusions or distrusted the compensation system were all associated with markedly higher vaccine hesitancy. In contrast, knowledge level showed a negative association: higher scores in AEFI-related knowledge and compensation mechanism knowledge corresponded to lower vaccine hesitancy. Socioeconomic factors also mattered: compared with the lowest-income group, guardians from higher-income households displayed relatively lower vaccine hesitancy, indicating that economic capacity can, to some extent, buffer public concerns about vaccine risks. These findings support the 3C model of vaccine hesitancy, which emphasizes the importance of confidence, complacency, and convenience in vaccination decisions [20,23]. In addition, this study found that guardians who participated in parenting classes had significantly lower vaccine hesitancy, highlighting the important role of health education interventions in reducing vaccine hesitancy. This is consistent with behavioral science research, which shows that targeted informational interventions can effectively improve vaccine attitudes and vaccination behavior [21]. Meanwhile, higher vaccine hesitancy was observed in rural areas and among low-income groups, suggesting persistent disparities in health resource accessibility and information access [17].

Based on these findings, this study carries both practical and theoretical implications. Practically, targeted health education should be strengthened to improve public understanding of AEFI causality and coincidental illnesses; transparency of the AEFI compensation system should be enhanced via case dissemination to build public trust; health education coverage should be expanded to reach high-risk groups including low-income households, rural residents, and those with a history of vaccine hesitancy; and strategies for non-NIP vaccines should be optimized by combining financial subsidies and risk communication to reduce economic and psychological barriers. These measures may help support vaccine confidence and strengthen the national immunization program efforts. Theoretically, this study extends the 3C model by incorporating China-specific institutional factors. The findings complement previous studies based on the 3C model by highlighting the potential relevance of compensation awareness and institutional trust in understanding vaccine hesitancy. However, the cross-sectional design does not allow conclusions regarding causal pathways or mediating relationships among these factors.

From a public health perspective, several implications emerge. First, risk communication strategies should place greater emphasis on explaining the causal assessment of AEFI, including the concept of coincidental illnesses. Second, improving public awareness and transparency of compensation mechanisms may help build trust in vaccination systems. Third, targeted interventions should focus on vulnerable groups, including rural populations and low-income households. Finally, for non-NIP vaccines, strategies addressing both perceived risk and financial barriers may be necessary to improve uptake.

## 5. Strengths and Limitations

This study has several strengths, including a large sample size, multistage random sampling, and the simultaneous assessment of AEFI knowledge, compensation awareness, institutional trust, and vaccine hesitancy. However, several limitations should be acknowledged. First, the cross-sectional design precludes causal inference and limits interpretation of the relationships among knowledge, trust, and vaccination attitudes. Second, all measures were self-reported and may be subject to recall bias and social desirability bias. Third, although Chongqing is broadly representative of western China, the findings may not be fully generalizable to other regions. Finally, subgroup analyses were not performed, and differences in knowledge classification criteria across studies may affect the comparability of findings.

## 6. Conclusions

Vaccine hesitancy among caregivers in Chongqing was associated with cognitive, economic, and institutional factors. Public health strategies should prioritize improving risk communication on AEFI causality—particularly the distinction between vaccine reactions and coincidental illnesses—and enhancing the transparency and accessibility of compensation policies to strengthen public trust and reduce vaccine hesitancy.

## Figures and Tables

**Table 1:** Awareness rates of AEFI and insurance compensation knowledge among child caregivers in Chongqing, 2025 (*n* = 3497).

Relevant Knowledge	Correct Answer	Number of Correct Answers	Correct Rate (%)
Awareness of AEFI knowledge			
1. Is it a common adverse reaction after vaccination to experience mild fever, redness, swelling and pain at the injection site within a short period after vaccination?	Right	2955	84.5
2. Should one stay for observation for at least 30 min after vaccination?	Right	3418	97.7
3. Can adverse events occur after the administration of every vaccine?	Right	2128	60.9
4. If your child has any discomfort symptoms such as fever, cough, runny nose or diarrhea recently, should you report them to the vaccination doctor during registration?	Right	3294	94.2
5. If a vaccine recipient is in the incubation stage of a certain disease and develops the disease shortly after vaccination, does it belong to a vaccine reaction? *	Wrong	900	25.7
Awareness of insurance compensation knowledge			
1. Will insurance companies compensate for vaccination-related AEFI in accordance with relevant policies?	Right	2865	81.9
2. Does the compensation insurance cover basic insurance and supplementary insurance, which are purchased for children by the government and guardians respectively?	Right	2589	74.0
3. Can medical expenses and other compensations be obtained if an adverse reaction following immunization occurs after receiving NIP vaccines?	Right	2581	73.8
4. If a disease occurring after vaccination is investigated and confirmed to be unrelated to immunization, can its treatment costs be compensated through basic insurance?	Wrong	1999	57.2
5. After guardians purchase supplementary insurance for AEFI for children, can they receive additional compensation such as loss of income?	Right	1956	55.9
6. Can supplementary insurance for adverse events following immunization provide compensation for adverse reactions caused by non-NIP vaccines?	Right	2180	62.3
7. Can supplementary insurance for AEFI cover the medical expenses of coincidental illnesses occurring after vaccination?	Right	2058	58.9
8. Is the coverage period for the first purchase of supplementary insurance for adverse events following immunization 0 to 6 years of age?	Right	2217	63.4

Note: * This question aims to assess awareness of ‘coincidental illnesses’—a common scenario where the onset of a preexisting disease coincides with vaccination timing, but the disease is not causally related to the vaccine.

**Table 2:** Multivariable linear regression analysis of factors associated with vaccine hesitancy scores among child caregivers in Chongqing, 2025 (*n* = 3497).

Variable	Group	N	Multivariate Analysis	
			*β*	95% CI
Survey region	Urban	2487	0.00	
	Rural area	1010	0.37	0.05~0.69
Total monthly family income (k yuan)	<3	275	0.00	
	3–9	2379	−0.99	−1.53~−0.46
	10–19	744	−1.08	−1.67~−0.49
	≥20	99	−1.26	−2.28~−0.28
Any guardian attended parent immunization classes	Yes	2064	0.00	
	No	1433	0.54	0.27~0.80
Child age in years	0–2	2024	0.00	
	3–5	1056	−0.56	−0.84~−0.27
	6	417	−0.28	−0.68~0.13
History of vaccine hesitancy	No	2319	0.00	
	Yes	1178	1.65	1.31~1.99
History of refusal to vaccinate	No	2761	0.00	
	Yes	736	1.04	0.65~1.43
AEFI knowledge score	0–2	349	0.00	
	3–5	3148	−1.14	−1.41~−0.81
Insurance compensation knowledge score	0–4	1223	0.00	
	5–8	2274	−2.06	−2.54~−1.58
Perception of childhood vaccine safety	Very safe	2974	0.00	
	Somewhat unsafe/unsafe	523	2.30	1.92~2.68
Acceptance of AEFI diagnosis by investigation panel	Yes	2346	0.00	
	No	1151	0.62	0.29~0.95
AEFI medical expenses	Out-of-pocket	749	0.00	
	Non-out-of-pocket	2748	−0.76	−1.07~−0.46
Approval of the current vaccine injury compensation mechanism	Yes	2858	0.00	
	No	639	1.38	1.01~1.75

## Data Availability

The data presented in this study are available on request from the corresponding author.

## Supplementary Materials

The following supporting information can be downloaded at: https://www.mdpi.com/article/10.3390/vaccines14070577/s1, Table S1: Participant characteristics and univariate associations with vaccine hesitancy.
