# Supplementary material for: Vaccine Hesitancy Among Caregivers in China: Associations of Misinterpreted AEFI, Compensation Awareness, and Institutional Trust
Source: Vaccines (Basel). 2026 Jun 30;14(7):577. doi: 10.3390/vaccines14070577 (PMC13417089; doi:10.3390/vaccines14070577)
Supplement: Supplementary file 1 [file vaccines-14-00577-s001.zip › vaccines-4387199-supplementary.pdf]

Supplementary Table S1

Participant characteristics and univariate associations with vaccine hesitancy

Variable	Group	N	Vaccine hesitancy			Univariate analysis	
			n	%	Mean±SD	*t/F*	*P*
Survey region	Urban	2487	222	8.9	23.11±4.46	-3.14*	0.002
	Rural area	1010	79	7.8	23.57±3.74		
Parent gender	Male	1790	156	8.7	23.28±4.31	0.24	0.626
	Female	1707	145	8.5	23.21±4.23		
Parent Age in years	＜30	1181	111	9.4	23.35±4.21	0.61	0.543
	30-39	1966	161	8.1	23.17±4.37		
	⩾40	350	29	8.2	23.27±3.87		
Occupation	Government/enterprise staff	1767	143	8.0	22.82±4.36	11.89	＜0.001
	Commercial/service staff	260	23	8.8	23.48±4.44		
	Agriculture/migrant workers	389	29	7.4	23.73±3.93		
	Unemployed	1081	106	9.8	23.69±4.11		
Primary caregiver for childhood immunization	Parents	2991	257	8.6	23.24±4.30	0.05	0.827
	Grandparents	506	44	8.7	23.28±4.06		
Total monthly family income （RMB yuan）	＜3000	275	37	13.5	24.19±4.03	15.38*	0.002
	3000-9000	2379	176	7.4	23.19±4.11		
	10000-19000	744	74	10.0	23.10±4.78		
	⩾20000	99	14	14.1	22.93±4.32		
Parents working outside for a long time	Yes	1025	92	8.9	23.47±4.18	4.29	0.038
	No	2472	209	8.5	23.15±4.30		

Any guardian attended parent immunization classes	Yes	2064	154	7.5	22.73±4.46	-8.89*	< 0.001
	No	1433	147	10.3	23.98±3.86		
Child age in years	0-2	2024	197	9.7	23.53±4.24	22.28*	< 0.001
	3-5	1056	77	7.3	22.75±4.24		
	6	417	27	6.5	23.08±4.36		
Relationship with the Child	Father	2798	226	8.9	23.24±4.22	0.19*	0.911
	Mother	471	61	13.0	23.27±4.82		
	Grandparent	228	14	6.1	23.23±3.58		
Only child in family	Yes	1703	161	9.5	23.07±4.49	-2.26*	0.024
	No	1794	140	7.8	23.40±4.04		
Mothers' education	Primary school or below	169	15	8.9	23.76±4.17	8.02	< 0.001
	Secondary school or junior college	1873	176	9.4	23.45±4.18		
	Bachelor's degree or above	1455	109	7.5	22.91±4.36		
Underlying Medical Conditions	No	3463	295	8.5	23.23±4.26	1.35	0.245
	Yes	34	6	17.7	24.09±4.81		
Child's AEFI history	No	3083	253	8.2	23.15±4.31	-3.71*	< 0.001
	Yes	414	48	11.6	23.92±3.89		
History of vaccine hesitancy	No	2319	137	5.9	22.31±4.23	19.77*	< 0.001
	Yes	1178	164	13.9	25.07±3.71		
History of refusal to vaccinate	No	2761	198	7.2	22.72±4.23	15.47*	< 0.001
	Yes	736	103	14.0	25.22±3.81		

AEFI knowledge score	0-2	349	83	23.8	25.49±4.48	111.26	< 0.001
	3-5	3148	218	6.9	22.99±4.17		
Insurance compensation knowledge score	0-4	1223	168	13.7	24.18±4.23	92.22	< 0.001
	5-8	2274	133	5.9	22.74±4.21		
Perception of childhood vaccine safety	Very safe	2974	165	5.6	22.64±4.06	-22.59*	< 0.001
	Somewhat unsafe/unsafe	523	136	26.0	26.69±3.73		
Acceptance of AEFI diagnosis by investigation panel	Yes	2346	122	5.2	22.64±4.21	-12.30*	< 0.001
	No	1151	179	15.6	24.28±4.12		
Supplementary insurance for AEFI	Purchased	807	55	6.8	22.83±4.24	9.63	0.002
	Not purchased	2690	246	9.1	23.36±4.27		
Other commercial insurance for AEFI	Purchased	1296	102	7.9	23.01±4.35	-2.43*	0.015
	Not purchased	2201	199	9.0	23.38±4.21		
AEFI medical expenses	Out-of-pocket	749	84	11.2	24.19±4.47	48.02	< 0.001
	Non-out-of-pocket	2748	217	7.9	22.98±4.17		
Approval of the current vaccine injury compensation mechanism	Yes	2858	153	5.4	22.69±4.08	288.16	< 0.001
	No	639	148	23.2	25.73±4.20		

Note: *Levene test showed unequal variances, so Welch's ANOVA or t′ test was used.
